# Mutations and Deregulation of Ras/Raf/MEK/ERK and PI3K/PTEN/Akt/mTOR Cascades Which Alter Therapy Response

**DOI:** 10.18632/oncotarget.652

**Published:** 2012-09-20

**Authors:** James A. McCubrey, Linda S. Steelman, William H. Chappell, Stephen L. Abrams, Giuseppe Montalto, Melchiorre Cervello, Ferdinando Nicoletti, Paolo Fagone, Grazia Malaponte, Maria C. Mazzarino, Saverio Candido, Massimo Libra, Jörg Bäsecke, Sanja Mijatovic, Danijela Maksimovic-Ivanic, Michele Milella, Agostino Tafuri, Lucio Cocco, Camilla Evangelisti, Francesca Chiarini, Alberto M. Martelli

**Affiliations:** ^1^ Department of Microbiology and Immunology, Brody School of Medicine at East Carolina University, Greenville, NC, USA; ^2^ Department of Internal Medicine and Specialties, University of Palermo, Palermo, Italy; ^3^ Consiglio Nazionale delle Ricerche, Istituto di Biomedicina e Immunologia Molecolare “Alberto Monroy”, Palermo, Italy; ^4^ Department of Biomedical Sciences, University of Catania, Catania, Italy; ^5^ Department of Medicine, University of Göttingen, Göttingen, Germany; ^6^ Department of Immunology, Instititue for Biological Research “Sinisa Stankovic”, University of Belgrade, Belgrade, Serbia; ^7^ Regina Elena National Cancer Institute, Rome, Italy; ^8^ Sapienza, University of Rome, Department of Cellular Biotechnology and Hematology, Rome, Italy; ^9^ Department of Biomedical and Neuromotor Sciences, Università di Bologna, Bologna, Italy; ^10^ Institute of Molecular Genetics, National Research Council-Rizzoli Orthopedic Institute, Bologna, Italy

**Keywords:** Targeted Therapy, Therapy Resistance, Mutations, Raf, Akt, PI3K, mTOR

## Abstract

The Ras/Raf/MEK/ERK and PI3K/PTEN/Akt/mTOR cascades are often activated by genetic alterations in upstream signaling molecules such as receptor tyrosine kinases (RTK). Certain components of these pathways, *RAS, NF1, BRAF, MEK1, DUSP5, PP2A, PIK3CA, PIK3R1, PIK3R4, PIK3R5, IRS4, AKT, NFKB1, MTOR, PTEN, TSC1*, and *TSC2* may also be activated/inactivated by mutations or epigenetic silencing. Upstream mutations in one signaling pathway or even in downstream components of the same pathway can alter the sensitivity of the cells to certain small molecule inhibitors. These pathways have profound effects on proliferative, apoptotic and differentiation pathways. Dysregulation of components of these cascades can contribute to: resistance to other pathway inhibitors, chemotherapeutic drug resistance, premature aging as well as other diseases. This review will first describe these pathways and discuss how genetic mutations and epigenetic alterations can result in resistance to various inhibitors.

## INTRODUCTION

This review is an updated and expanded version of a previous review on this topic [1]. This current review now discusses some of the types and classes of mutations which occurs in these pathways and their biochemical importance in terms of therapy. We will focus on the recent advancements in elucidating the roles of the Ras/Raf/MEK/ERK and Ras/PI3K/Akt/mTOR pathways and the types and classes of mutations which occur in these pathways. Since the discovery of the *RAS*, *RAF*, *MEK*, *PIK3CA*, and *AKT* oncogenes and *NF1*, *DUSP5*, *PP2A*, *PTEN*, *TSC1* and *TSC2* tumor suppressor genes, the Ras/Raf/MEK/ERK and Ras/PI3K/PTEN/Akt/mTOR signaling cascades have been extensively investigated with the ultimate goal of determining how these genes become activated/inactivated and whether it is possible to suppress their activity in cancer and other growth-related diseases [1-6]. Furthermore these pathways are also frequently implicated in the resistance and sometimes sensitivity to therapy [1-6]. Research has also resulted in the development of inhibitors that target critical components of these pathways with the ultimate goal to increase patient survival or in some cases to prevent or impede the development of other diseases (*e.g*., obesity, diabetes and premature aging) [7-9].

Before we discuss the Ras/Raf/MEK/ERK and Ras/PI3K/PTEN/Akt/mTOR signaling cascades, it is important to define some genetic terms as they are critical to understanding the importance of these pathways and the classes of genes and mutations that occur in components of these cascades. We briefly discuss certain classes of genes which play key roles in the development of cancer. Caretaker genes are involved in genomic stability and normally function to suppress the mutation rate [10]. Caretaker mutations occur mainly in tumor suppressor genes, such as *TP53* (p53) and *PTEN*. *TP53* and *PTEN* are caretaker genes. Caretaker genes help maintain the integrity of the genome.

Gatekeeper genes directly regulate cell growth and their loss can lead to tumorigenesis. They encode critical proteins which can regulate growth or the induction of apoptosis. Many genes fall into this class including: *MAPK3/MAPK1* (ERK1/ERK2), *TP53, PTEN, NF1, TSC1 TSC2, MTOR* (mTOR), *EIF4E* (eIF4E). Obviously some genes can fall into multiple classifications [11].

The concept of a driver mutation is very important in cancer. If the driver mutation can be successfully targeted that may lead to elimination of the cancer. This is a mutation that is statistically enriched in a particular cancer and usually thought to be one of the first events in the malignant transformation of those particular cells to cancer cells [12]. Examples of driver mutations in these two pathways include *RAS*, *NF1*, *BRAF*, *MEK1*, *PIK3CA* (PI3K), and *PTEN*.

A second class of mutations is passenger mutations [6]. Passenger mutations may occur by different mechanisms. Passenger mutations may occur upon genomic deletion of the region of the chromosome which contains the driver mutation [6]. This has been observed in glioblastoma which have the glycolytic gene enolase 1 (*ENO1*) gene deleted as it is in the neighborhood of the 1p36 tumour-suppressor locus [6]. *ENO1* is a member of a gene family and there are two other *ENO* genes. Normally, the cell can survive in the presence of *ENO1* deletion, however, if *ENO2* is silenced, the cancer cell with the *ENO1* deletion dies. This provides a selective approach to kill cancer cells, illustrating the significance of identifying passenger mutations.

Next we discuss types of mutations which can result in therapeutic resistance. Gatekeeper mutations often occur in genes (often protein kinases) in either the inhibitor binding site or in the ATP binding site of the protein. They are detected in *BRAF, ERK, BCRABL*, and epidermal growth factor receptor (*EGFR*) and can mediate resistance to small molecule inhibitors as that is often where they bind and inhibit activity. They have also been detected in *PIK3CA* but not necessarily in the hot spot locations [13,14]. Hot spot locations are regions of the gene where mutations are most frequently detected and they can confer a biochemical advantage to the cells which allows abnormal growth.

A synthetic lethal mutation refers to a mutation that occurs in a second gene and results in the death of the cell. This terminology was coined by yeast geneticists. Synthetic lethal screening has resulted in the elucidation of how certain gene products interact with other gene products forming biochemical pathways [15,16]. For example, when there is an activated oncogene or inactivated tumor suppressor gene present in a cell which frequently leads to the abnormal proliferation of the cells, a synthetic lethal mutation may occur at a second gene which results in the death of the transformed cell [17-19]. In essence, there is the loss of a biochemical interaction between the mutant oncogene or tumor suppressor gene and the second gene and the cell dies. Hence the second mutation is referred to as synthetic lethal. In terms of the Ras/Raf/MEK/ERK pathway, which proliferates in response to mutant *KRAS*, silencing of genes such as voltage-dependent anion channel (*VDAC1*), serine/threonine kinase 33 (*STK33*), TANK- binding kinase 1 (TBK) or polo-like kinase-1 (*PLK1*) results in synthetic lethal interactions [15]. Synthetic lethal interactions are frequently identified by screening siRNA or shRNA libraries. In the PI3K/PTEN/Akt/mTOR pathway, a synthetic lethal interaction is observed in renal cell carcinoma (RCC) cells which lack the von Hippel–Lindau tumor suppressor protein (*VHL*) as treatment of the cells with rapamycin, an inhibitor of mTORC1 which the tumor cells are dependent on, results in death [15,16].

Lineage-specific mutations occur in genes which are abnormally expressed in certain types of cancers. In certain cell types, the cells become addicted to a lineage-specific gene as well as the mutant oncogene(s). An example is observed in melanoma cells which have mutant *BRAF*. These cells often have increased expression of the microphthalmia-associated transcription factor (*MITF*) which is believed to allow the survival of cells of the melanocyte lineage. *MITF* is sometimes amplified in certain subsets of melanoma cells and cooperates with mutant *BRAF* to regulate melanoma proliferation. In normal melanocytes, MITF induces cell cycle arrest, whereas in melanoma cells, mutant B-Raf may stimulate *MITF* transcription while this stimulation of transcription does not occur in normal melanocytes [20].

Oncogene-addiction is a widely-used term to describe the transformed cells addiction to a particular gene or pathway [20-27]. The transformed cells frequently contain a mutation at a particular oncogene, or correspondingly, inactivation of a tumor suppressor gene. The cells become addicted to the consequences of that mutation and grow under conditions where a normal cell would not persist [22]. Many malignant melanoma cells become addicted to mutant *BRAF* for proliferation [20]. Likewise either mutation of *PIK3CA* or silencing of *PTEN* and subsequent activation of Akt is a frequent form of oncogene addiction in many tumor types [1,2,20-27].

Oncogene bypass occurs when a cell bypasses the signal transduction component it normally depended upon for survival [4]. This has been observed in certain cells which were normally dependent upon EGFR for survival, however, when upon exposure to an EGFR inhibitor, cells emerged which displayed amplification of another oncogene, the MET oncogene (MNNG-HOS transforming gene) which allowed the growth of the cells in the presence of the EGFR inhibitor [22].

Kinase switching is a similar event. An example is when cells with the *BRAF* V600E mutation were cultured in the presence of the B-Raf inhibitor SB-590885, inhibitor-resistant cells arose which utilized the related Raf-1 and A-Raf isoforms [28]. The genetic mechanisms for oncogene bypass and kinase switching as well as many of the changes in inhibitor-resistant cells are complicated and may result from the outgrowth of a minority of the cells present in the original tumor or cell line.

Oncogenic shock is a term that is used to describe the biochemical consequences of inhibiting the oncogene. Interestingly, it has been observed that upon inactivation of the oncogene responsible for survival, the pro-survival and pro-apoptotic signals decay at different rates. In absence of the oncogene responsible for the oncogene addiction phenotype, the pro-survival signals decay more rapidly than the pro-apoptotic signals. This has led to the concept of oncogenic shock and provides the basics for the success of certain inhibitors in suppressing the growth of oncogene-transformed cells [25]. Oncogenic shock may be connected with the translation of “weak mRNAs” which are regulated by the mTOR complex 1 (mTORC1) (see below). Both the Ras/Raf/MEK/ERK and PI3K/PTEN/Akt/mTOR pathways interact to regulate the activity of the mTORC1 complex. The half-lifes of proteins such as Akt and ERK are very short (within minutes), while the half-lifes of pro-apoptotic signals are much longer (hours). The decreased activity of Akt and ERK proteins will have a direct effect on the translation of weak mRNAs which often encode growth factors and other important proteins regulating cell growth (*e.g*., c-Myc). This is one reason why targeting the Ras/Raf/MEK/ERK and PI3K/PTEN/Akt/mTOR pathways has such profound effects on cell growth.

Non-oncogene addiction is a more recently devised term to describe the addiction of a cell on another gene which is not an oncogene *per se* [26]. For example, rapamycin and modified rapamycins (rapalogs) target mTORC1 which is not normally considered an oncogene, but the cells are dependent upon the mTORC1 complex for their survival. RCC which lack the pVHL tumor suppressor protein exhibit non-oncogene addiction [27]. Now that we have discussed some general genetic terms, we can discuss in more detail the Ras/Raf/MEK/ERK and PI3K/PTEN/Akt/mTOR pathways.

### The Ras/Raf/MEK/ERK Pathway

**Figure 1:**
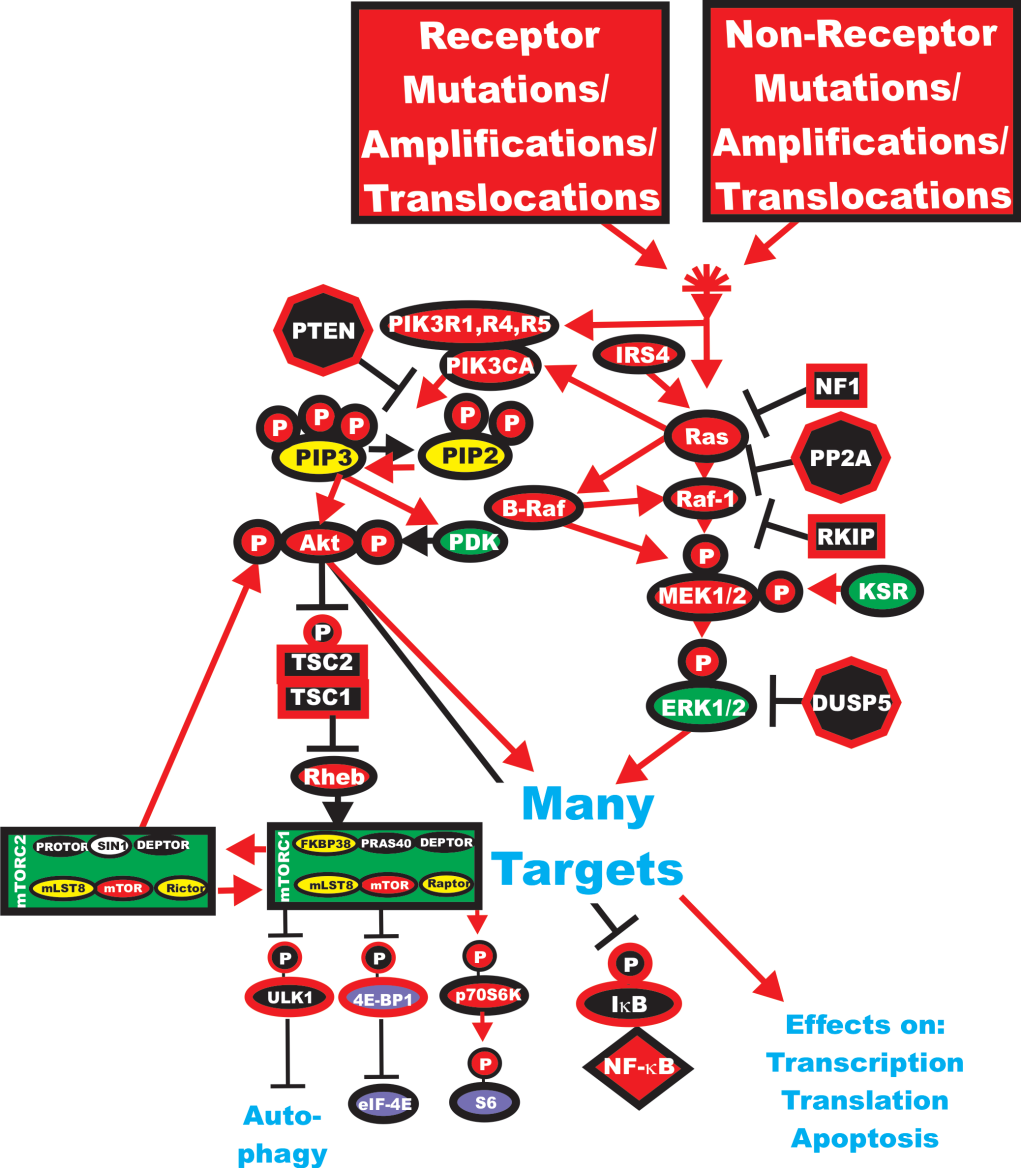
Activation of the Ras/Raf/MEK/ERK and Ras/PI3K/PTEN/Akt/mTOR Pathways by Genetic Mutations Activation of the Ras/Raf/MEK/ERK and Ras/PI3K/PTEN/Akt/mTOR pathways can occur by upstream mutations in growth factor receptors or by mutations in upstream kinases and coupling molecules. In addition, intrinsic members of the two pathways are frequently mutated in human cancer. Genes in the Ras/Raf/MEK/ERK and Ras/PI3K/PTEN/Akt/mTOR pathways that have activating mutations detected in human cancer and proliferative diseases are indicated in red ovals. Four mutations have been detected in the RHEB gene in 903 samples present in the Sanger Institute COSMIC database. Four mutations have been observed in the p70S6K gene (*RBS6KB1*) out of 1047 samples examined (Sanger Institute COSMIC database. Also loss of tumor suppressor genes such as *PTEN*, *PP2A*, *NF1 DUSP5*, *TSC1*, *TSC2* can contribute to activation of certain pathways. These are indicated in black octagons if they are phosphatases or black squares if they are coupling molecules. Other kinases not frequently mutated in human cancer are indicated in green symbols. PIP2 and PIP3 are indicated in yellow ovals. The 4E-BP1and eIF-4E proteins are indicated in purple ovals. mTORC1 phosphorylates the unc-51-like kinase 1 (ULK1) which results in the suppression of autophagy. ULK1 is indicated in a black oval. The mTORC1 inhibitor prevents phosphorylation of ULK1 and autophagy can occur. Red arrows indicate activating events in pathways. Black arrows indicate inactivating events in pathway. Activating phosphorylation events are depicted in red circles with Ps with a black outlined circle. Inactivating phosphorylation events are depicted in black circles with Ps with a red outlined circle.

Usually signaling commences upon ligation of a growth factor/cytokine/interleukin/mitogen (ligand) to its cognate receptor at the cell surface. This event can result in the activation of many downstream signaling cascades including the Ras/Raf/MEK/ERK and Ras/PI3K/PTEN/Akt/mTOR pathways. These cascades can further transmit their signals to the nucleus to control gene expression, to the translational apparatus to enhance the translation of “weak” mRNAs, to the apoptotic machinery to regulate apoptosis or to other events involved in the regulation of cellular proliferation (for example, interactions with the p53 pathway to regulate cell cycle progression). Regulation of the Ras/Raf/MEK/ERK and Ras/PI3K/PTEN/Akt/mTOR pathways is mediated by a series of kinases, phosphatases, GTP:GDP exchange and scaffolding proteins. There are also many tumor suppressor proteins which interact with these cascades which frequently serve to fine tune or limit activity (*e.g*., PTEN, RKIP, PP2A, DUSP5, DUSP6, TSC1, TSC2). Mutations occur in many of the genes in these pathways leading to uncontrolled regulation and aberrant signaling [5,28-32]. Certain of these tumor suppressor genes can be regulated by oncogenic micro (mi) RNAs [33]. An overview of the effects of mutations and the activation of the Ras/Raf/MEK/ERK and Ras/PI3K/PTEN/Akt/mTOR signaling pathways and how they interact is presented in Figure 1. In this review, we will point out which genes are abnormally expressed in human cancer to illustrate the importance of these genes and pathways.

Following stimulation of a growth factor receptor (GFR), a Src homology 2 domain containing protein (Shc) adaptor protein becomes associated with the C-terminus of the activated GFR, *e.g*., EGFR, insulin like growth factor-1 receptor (IGF-1R), vascular endothelial growth factor receptor (VEGFR) and many others [1-3, 34-38]. *EGFR* mutations can contribute to transformation of multiple cell lineages and these alterations are considered driver mutations

Shc recruits the growth factor receptor-bound protein 2 (Grb2) protein and the son of sevenless (SOS) homolog protein [a guanine nucleotide exchange factor (GEF)], resulting in the loading of the membrane-bound GDP:GTP exchange protein (GTPase) Ras with GTP [1,2]. *RAS* is frequently mutated in many diverse human cancers. *RAS* mutations are often driver mutations. GEFs promote Ras activation by displacing GDP from Ras which leads to GTP binding.

Ras activation is suppressed by the GTPase activating proteins (GAPs) that stimulate the GTPase activity of Ras. There are two prominent GAP proteins, p120GAP and NF1. *NF1* is a tumor suppressor gene and has both driver and gatekeeper gene functions. Germline mutations at *NF1* lead to neurofibromatosis [29].

Ras can also be activated by GFRs, such as insulin receptor (IR), via intermediates like insulin receptor substrate (IRS) proteins that bind Grb2 [34]. *IRS4* has recently been documented to be mutated in melanoma [5]. Ras:GTP then recruits the serine/threonine (S/T) kinase Raf to the membrane where it becomes activated, likely via a Src-family tyrosine (Y) kinase [1,2]. Recently Ras-mediated Raf-1 activation has been shown to be dependent on calcium/calmodulin-dependent protein kinase II (CaMK-II) which phosphorylates Raf-1 at S338 in some experimental stimulation conditions [EGF, fetal bovine serum (FBS) treatment]. This dependency does not appear to occur with regards to B-Raf activation [39].

Both *RAS* and *RAF* are members of multi-gene families and there are three Ras members (*KRAS*, *NRAS* and *HRAS*) [1-4] and three *RAF* members [*BRAF*, *RAF1* (a.k.a c-Raf) and *ARAF*) [1-3]. *BRAF* is frequently mutated in melanomas and certain other cancers and these mutations are frequently driver mutations [40].

Raf-1 can be regulated by dephosphorylation by the protein serine/threonine phosphatase 2A (PP2A) and others [41,42]. PP2A has been reported to positively and negatively regulate Raf-1. *PP2A* is also considered a tumor suppressor gene and has gatekeeper gene functions [43].

Raf phosphorylates and activates the mitogen-activated protein kinase kinase-1 (MEK1) (a dual specificity kinase (T/Y) on S/T residues [1-4]). Other proteins such as kinase suppressor of Ras (KSR) have recently been shown to phosphorylate MEK1 [44-48]. KSR has scaffolding properties and interacts with Raf, MEK and ERK which regulate ERK activation [44-48]. KSR can form dimers with various Raf proteins which alter the effects of Raf inhibitors. KSR1 competes with Raf-1 for Raf inhibitor-induced binding to B-Raf which decreases the normal ERK activation observed after Raf-inhibitor treatment [47].

MEK1 phosphorylates extracellular signal regulated kinases 1/2 (ERK1 and 2) at specific T /Y residues [1-4]. *MEK1* was originally not thought to be mutated frequently in human cancer. However, recent large scale mutation screening studies and studies aimed at determining mechanisms of resistance to small molecule inhibitors have observed that *MEK1* is mutated in certain human cancers and also is mutated in certain inhibitor-resistant cells. *MEK1* is also considered to be a driver oncogene in certain cancers [49]. Rac (Ras related gene) and p21-activating kinases (PAK) can also regulate MEK/ERK activation [50,51].

Activated ERK1 and ERK2 S/T kinases phosphorylate and activate a variety of substrates, including p90 Ribosomal six kinase-1 (p90^Rsk1^) and this pathway has been implicated in cancer progression [1-3]. ERK1/2 are considered by some as gatekeeper genes. ERK also phosphorylates MAPK signal integrating kinases (Mnk1/2) which can in turn phosphorylate (eukarytotic translation initiation factor 4E) eIF4E, a key protein involved in the translation of difficult mRNAs [1-3]. *EIF4E* is considered to be a gatekeeper gene.

p90^Rsk1^ can activate the cAMP response element binding protein (CREB) transcription factor as well as proteins involved in regulation of protein translation (*e.g*., Mnk-1, p70 ribosomal S6 kinase (p70S6K), eukaryotic translation initiation factor 4B, (eIF4B), and ribosomal protein S6 (rpS6) [52].

**Figure 2:**
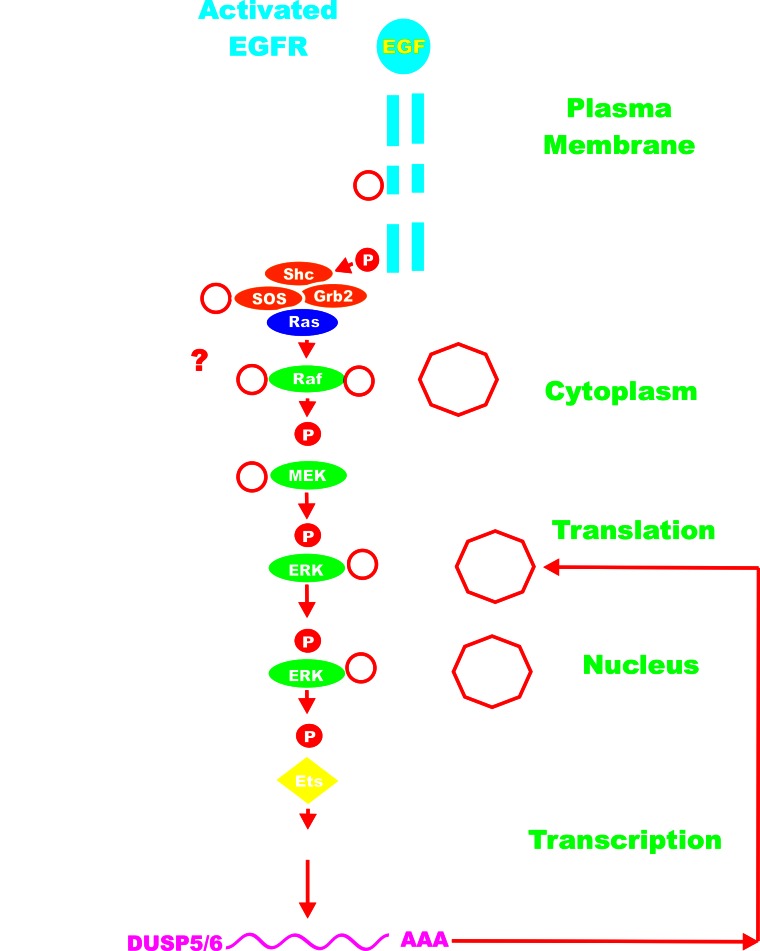
Regulatory Loops in the Ras/Raf/MEK/ERK Pathway ERK can phosphorylate members of the Ras/Raf/MEK/ERK pathway and even upstream EGFR. Sometimes these phosphorylation events mediated by ERK can inhibit the activity of the phosphorylated molecule. ERK can also phosphorylate Ets which can lead to transcription of the DUSP genes which in turn can inactivate ERK by dephosphorylation. PP2A can also suppress Raf activity by dephosphorylation. The activated EGFR is depicted in blue. Kinases are indicated by green ovals. Phosphatases are indicated by black octagons. Coupling molecules are indicated by orange ovals. Ras is indicated by a purple oval. The transcription factors Ets is indicated by a yellow diamond. The activated EGFR is depicted in blue. Red arrows indicate activating events in pathways. Black arrows indicate inactivating events in pathway. Activating phosphorylation events are depicted in red circles with Ps with a black outlined circle. Inactivating phosphorylation/dephosphorylation events are depicted in black circles with Ps with a red outlined circle.

The number of ERK1/2 substrates/targets is easily in the hundreds. These substrate/targets include different types of molecules including: other kinases, phosphatases, growth factor receptors, cytokines, cell cycle regulator proteins, transcription factors, or proteins involved in mRNA translation or apoptosis. Suppression of MEK and ERK can have profound effects on cell growth, inflammation and aging. Activated ERK can also phosphorylate “upstream” Raf-1 and MEK1 which alter their activity. Depending upon the site phosphorylated on Raf-1, ERK phosphorylation can either enhance [53] or inhibit [54] Raf-1 activity. In contrast, some studies have indicated that when MEK1 is phosphorylated by ERK, its activity decreases [55]. Recent studies indicate that ERK does not negatively feedback inhibit B-Raf [56]. ERK also phosphorylates SOS at multiples sites leading to the dissociation of SOS from GRB2 and preventing Ras activation [4, 57]. ERK can also phosphorylate EGFR and suppress its activity [58]. The dual specificity phosphatases (*DUSP*) (aka MKPs) are transcriptionally induced by ERK phosphorylation of transcription factors (*e.g*., Ets) [59]. The DUSPs serve as negative feedback regulators to suppress ERK activity. Some of the events induced by ERK phosphorylation are rapid, such as post-trasnlational modification, while other events require gene transcription and translation (*e.g*., ERK phosphorylation of Ets which induces transcription of DUSPs). The *DUSP*s are potentially tumor suppressor genes and DUSP mutations have been detected in various cancers [60]. An overview of the regulatory loops in the Ras/Raf/MEK/ERK pathway is presented in Figure 2.

The Raf/MEK/ERK pathway exhibits properties of a negative feedback amplifier (NFA). In essence, NFA signaling is similar in biological design to those used in electronic circuits. NFAs in electronic circuits optimize robustness, stabilization of signal and linearization of non linear signal amplification. These properties of the Raf/MEK/ERK NFA are important in determining activation kinetics, response to drugs and various other downstream effects of activated ERK [56].

Phosphorylation events induced by ERK serve to alter the stability and/or activities of the proteins. These examples of feed-back loops become important in consideration of whether to just target MEK or to target both Raf and MEK in various cancers. It is important that the reader realize that certain phosphorylation events can either inhibit or repress the activity of the affected protein. This often depends on the particular residue on the protein phosphorylated which can confer a different configuration to the protein or target the protein to a different subcellular localization that may result in proteasomal degradation or association with certain scaffolding proteins.

There are numerous scaffolding/chaperonin proteins which interact with various components of the Raf/MEK/ERK cascade (*e.g*., 14-3-3) [61], MEK partner-1 (MP-1) [62], heat shock protein-90 (HSP-90) [63], KSR [64] Raf kinase inhibitory protein (RKIP) [65]. Heat shock proteins such as HSP-90 are considered caretakers as they normally serve to protect the activity of client proteins [66, 67]. Mutations at *KRAS* will confer sensitivity to HSP-90 inhibitors such as geldanamycin, documenting the importance of HSP-90 in regulation of this pathway [68,69].

RKIP is also considered a metastasis suppressor gene in certain cancers and has gatekeeper and caretaker effects. Raf-1 activation by Ras has been shown to be dependent on the prohibitin protein, a ubiquitously expressed protein which may also serve as a chaperonin protein [70].

The regulation of ERK1/2 activity in the nucleus and cytoplasm is complex as the p38MAPK-alpha splice isoform Max-interacting protein (Mxi-2) can bind ERK1/2 and ensure its translocation into the nucleus and prevent both MAPK phosphatase-1 (MKP1) and DUSP5 from dephosphorylating ERK1/2 in the nucleus and not the cytoplasm. Most phosphatases will probably eventually be shown to be tumor suppressor genes. Upon Mxi-2 binding ERK1/2, enhanced ERK1/2 activity is detected in the nucleus. Mxi-2 prevents the dephosphorylation of ERK1/2 by MKP1 and DUSP5. This allows activated ERK1/2 to phosphorylate the transcription factor c-Myc and other critical substrates [71-73].

In the nucleus ERK can phosphorylate transcription factors, such as: E twenty-six-like transcription factor 1 (Elk-1), estrogen receptor (ER), Fos, globin transcription factor 1 (Gata-1), c-Myc, signal transducer activation of transcription 1 & 3 (STAT1 & 3) and others [1-3,55,74-76]. These transcription factors bind the promoters of many genes, including growth factor and cytokine genes that are important in promoting growth and preventing apoptosis of multiple cell types.

ERK can also phosphorylate and modulate the activity of the Twist, Snail, Slug, and Zeb1 transcription factors either directly or indirectly which can regulate cellular proliferation, survival and some can modulate epithelial mesenchymal transition (EMT) [77-92]. Phosphorylation of the transcription factors by ERK1/2, or in some cases the related MAPK, p38MAPK, prevents their ubiquitination and results in their stabilization and increased activity in the nucleus and ability to promote EMT [83-92].

In the nucleus, ERK can also phosphorylate mitogen and stress-activated protein kinases (MSKs) [93,94] which in turn can phosphorylate transcription factors such as activator transcription factor-1 (ATF-1) that is important in the regulation of many immediate early genes controlled by activating protein-1 (AP-1) [95]. The ternary complex factors (TCF) such as Elk-1, Sap-1 and Net are also phosphorylated by ERK which results in their activation [96,97]. The TCFs form complexes with serum responsive factor (SRF) and activate many genes through their serum responsive elements (SRE) in their promoter regions [98,99].

MSKs also phosphorylate many proteins involved in modulating chromatin structure including: Histone H3, and (high-mobility-group protein-14) HMG14 which can result in the transcription of immediate early genes after mitogens/growth factor stimulation [100]. ERK1/2 can phosphorylate many proteins critical for cytoskeletal structure/reorganization including: calpain (Capn) [101], focal adhesion kinase (FAK) [102], myosin light polypeptide kinase (MLCK) and paxillin-6 (Pax6) [103]. Sometimes phosphorylation by ERK of FAK can result in FAK dephosphorylation [104].

Thus the Ras/Raf/MEK/ERK pathway is important in determining cellular shape and mobility/invasion. Under certain circumstances, aberrant regulation of this pathway can contribute to abnormal cellular growth, mobility and invasion which may lead to many abnormalities including; autocrine transformation, drug resistance, senescence, premature aging, or metastasis [1,2,105-119].

**Figure 3:**
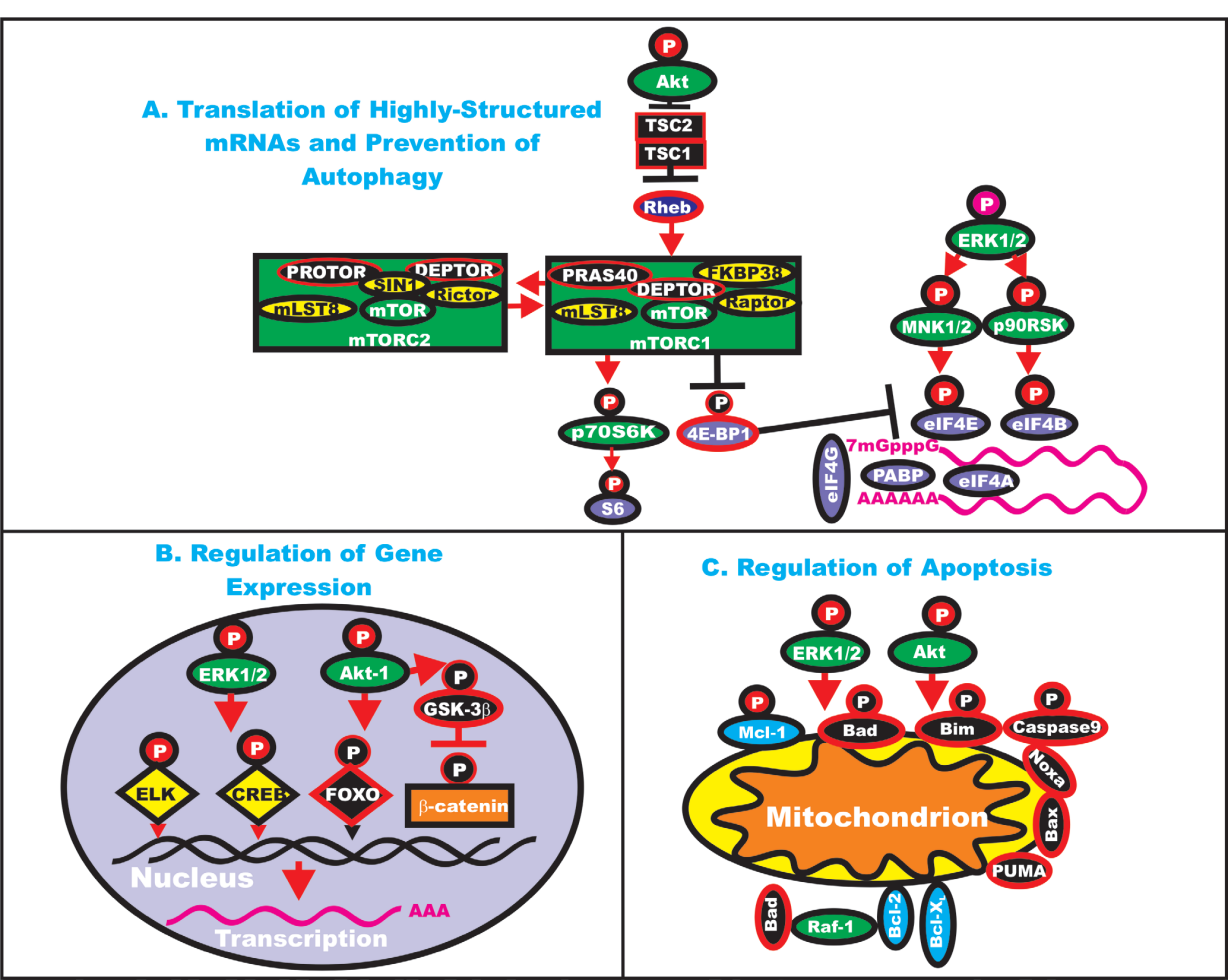
Effects of ERK and Akt on Regulatory Processes ERK and Akt can phosphorylate many targets which serve to regulate cell proliferation. In Panel A, some of the effects of ERK and Akt on the translation of highly structured mRNAs are indicated. Kinases are indicated in green ovals or rectangles (mTORC1 and mTORC2 complexes). TSC1 and TSC2 are indicated in black squares. Rheb is indicated in a dark blue oval. mTOR interacting proteins which positively regulate mTOR activity are indicated in yellow ovals. mTOR interactiving proteins which negatively regulate mTOR activity are indicated in black ovals. mRNA initiation factors and proteins associated with the ribosome are indicated in purple ovals. In Panel B, some of the effects on the regulation of gene expression by ERK and Akt phosphorylation are indicated. Transcription factors activated by either ERK or Akt phosphorylation are indicated in yellow diamonds. The Foxo transcription factor that is inactivated by Akt phosphorylation is indicated by a black diamond. Beta-catenin is indicated in an orange rectangle. In Panel C, some of the effects of ERK and Akt phosphorylation on apoptotic regulatory molecules are indicated. Molecules such as Mcl-1 which are anti-apoptotic and phosphorylated by ERK and Akt are indicated by blue ovals, other anti-apoptotic molecule are also indicated by blue ovals. Pro-apoptotic molecules are indicated by black ovals. Red arrows indicate activating events in pathways. Black arrows indicate inactivating events in pathway. Activating phosphorylation events are depicted in red circles with Ps with a black outlined circle. Inactivating phosphorylation events are depicted in black circles with Ps with a red outlined circle.

Thus the reader begins to understand how the Ras/Raf/MEK/ERK pathways can regulate the expression of many genes involved in the response to growth factors and mitogens. Furthermore many of the genes in this pathway, as well as other genes that regulate the activity of this pathway, have varying abilities to influence cancer development. They can sometimes be drivers of cancer development, gatekeeper or caretaker genes. An overview of the effects of the Ras/Raf/MEK/ERK and PI3K/PTEN/Akt/mTOR pathways on key regulatory pathways is presented in Figure 3.

### Mutations or Altered Expression of the Ras/Raf/MEK/ERK Pathway Components

In our previous reviews [1,3] we have discussed in detail the frequency of Ras mutations observed in human cancers. Ras mutations have been observed in approximately 20 to 30% of human cancers. Often point mutations are detected in *RAS* genes in cancer cells from patients which enhance Ras activity. Genome *RAS* amplification or overexpression of Ras, perhaps due to altered methylation of its promoter region, are also detected in some tumors [1]. The frequency of *KRAS* mutations is very high (~80%) in advanced pancreatic cancers [1]. Mutations in Ki-Ras will make cells sensitive to HSP90 inhibitors [68,69]. *BRAF* is mutated frequently in melanomas (50-70%) [5,120], papillary thyroid cancers (40%) [121], Langerhans’-cell histiocytosis (57%) [122]. *BRAF* is mutated to lesser extent (2-3%) in non small cell lung cancers (NSCLC), [123] and colo-rectal cancers (CRC) (8%) [124]. Recently *BRAF* has been observed to be frequently mutated (100%) in hairy cell leukemia. [125]. *BRAF* has been observed to be mutated in 8 of 199 patients (4%) with multiple myeloma and 4 of those were mutant at *BRAF* V600E [126]. Other B-ALL and peripheral B cell lymphomas have been observed to have low (>3%) frequencies of *BRAF* mutations, but none of these mutants produced the B-Raf V600E protein [127-130]. Similar mutations were not detected in the Tiacci *et al*. study with similar leukemias and lymphomas [125]. A recent study detected *BRAF* mutations in 2/55 (3.6%) of large B-cell lymphoma (DLBCL). The authors postulated that *BRAF* may be considered driver mutations for those DLBCL [131]. Cancer patients with the *BRAF* driver mutations are postulated to be sensitive to B-Raf inhibitors such as vemurafenib, dabrafenib, and GDC-0879.

Previously it was thought that the *MEK* and *ERK* genes were not frequently mutated in human cancer. More recent analysis has indicated that *MEK1* and *MEK2* are mutated in certain cancers (*e.g*., ovarian and lung cancers) and can be driver mutations [49, 131-134]. Mutations at *MEK1* are also important in governing the sensitivity/resistance of certain cells to Raf and MEK inhibitors and will be discussed in an accompanying review [134].

Upstream components of this pathway are also mutated or deregulated in human cancer [1-4]. Some common receptors which are altered in human cancer include EGFR, HER2, IGF-1R, PDGFR, VEGF, and FGFR2/3 [1-4].

### The Ras/PI3K/PTEN/Akt/mTOR Pathway

**Figure 4:**
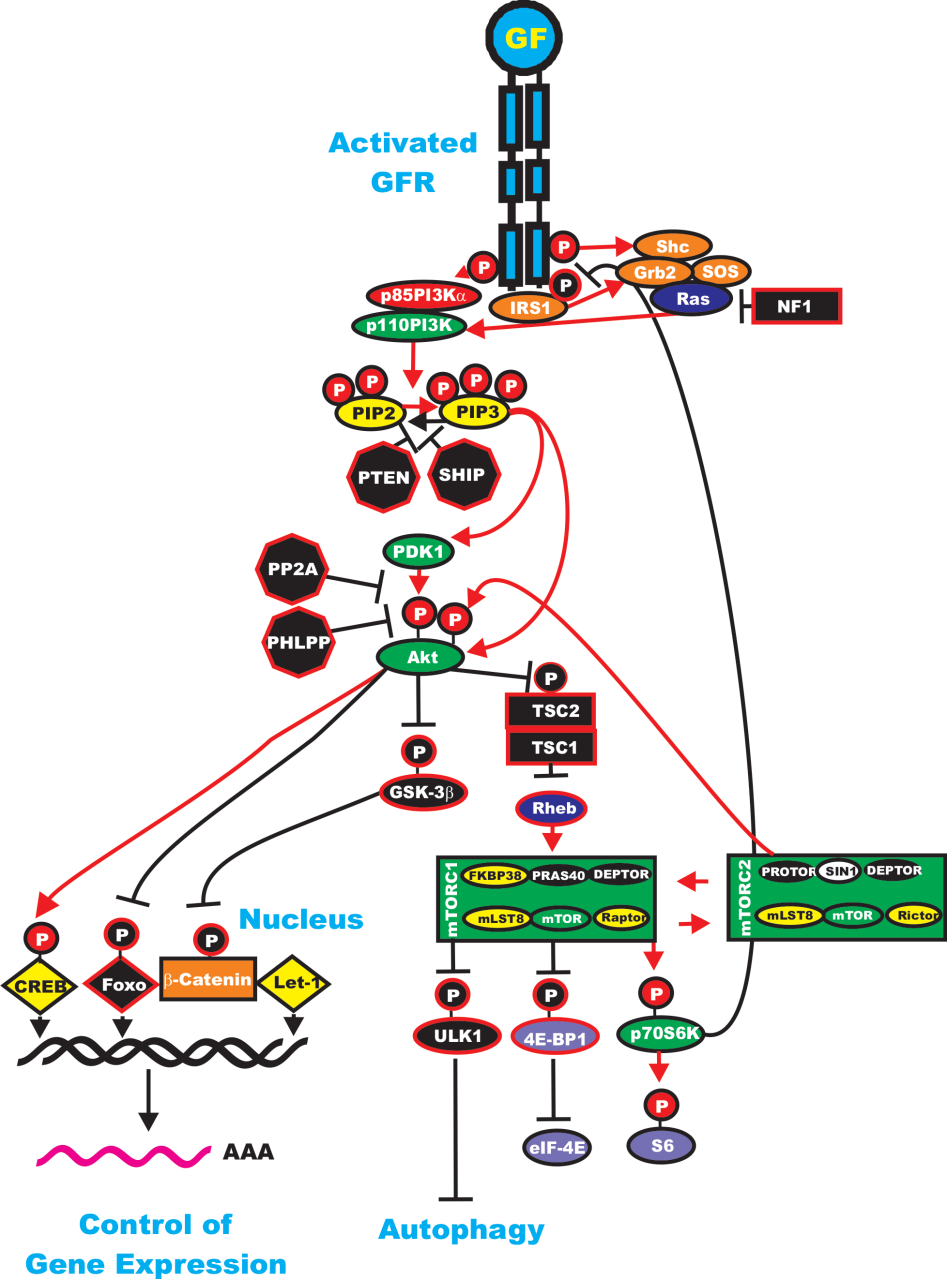
Regulatory Loops in the Ras/PI3K/PTEN/Akt/mTOR Pathway Some of the regulatory interactions in the Ras/PI3K/PTEN/Akt/mTOR Pathway are indicated. An activated growth factor receptor is indicated in blue. Ras and Rheb are indicated in dark blue ovals. IRS1 is indicated in an orange oval. Kinases are indicated in green ovals with the exception of GSK-3beta which is indicated in a black oval as it is inactivated by Akt phosphorylation. The p85 regulatory subunit of PI3K is indicated in a red oval. Phosphatases are indicated in black octagons. NF1, TSC1 and TSC2 are indicated in black squares. PIP2 and PIP3 are indicated in yellow ovals. Phosphatases are indicated in black octagons. mTOR interacting proteins which positively regulate mTOR activity are indicated in yellow ovals. mTOR interactiving proteins which negatively regulate mTOR activity are indicated in black ovals. Transcription factors activated by either ERK or Akt phosphorylation are indicated in yellow diamonds. The Foxo transcription factor that is inactivated by Akt phosphorylation is indicated by a black diamond. β-catenin is indicated in an orange rectangle. mRNA initiation factors and proteins associated with the ribosome are indicated in purple ovals. mTORC1 phosphorylates the unc-51-like kinase 1 (ULK1) which results in the suppression of autophagy. ULK1 is indicated in a black oval. The mTORC1 inhibitor prevents phosphorylation of ULK1 and autophagy can occur. Red arrows indicate activating events in pathways. Black arrows indicate inactivating events in pathway. Activating phosphorylation events are depicted in red circles with Ps with a black outlined circle. Inactivating phosphorylation events are depicted in black circles with Ps with a red outlined circle.

Phosphatidylinositol-3-kinase (PI3K) is a heterodimeric protein with an 85-kDa regulatory subunit and a 110-kDa catalytic subunit (*PIK3CA*) [1-3, 135-138]. *PIK3CA* is frequently mutated in certain cancers such as: breast, ovarian, colorectal, endometrial and lung [1,14,139] although its role as a driver mutation in these cancers remains controversial [140]. Recent studies have shown in the lung cancers with mutant *PIK3CA*, there are also mutations at other driver oncogenes, such as *EGFR*, *KRAS*, *BRAF*, *MEK*, and anaplastic lymphoma kinase (*ALK*) [141]. Recent studies in melanoma have indicated that some components of the PI3K pathway (*PTEN*, *MTOR*, *IRS4*, *PIK3R1*, *PIK3R4*, *PIK3R5* and *NFKB1*) are co-mutated in 17% of *BRAF* V600E mutant and 9% of *NRAS* mutant melanomas [5]. An overview of the Ras/PI3K/PTEN/Akt mTOR pathway and the regulator circuits is presented in Figure 4.

There are three classes of PI3K, each with distinct substrate specificity and lipid products: I, II, and III [135-138]. In mammals, class I PI3Ks are the best understood PI3Ks and are expressed in all cell types. To date, class I PI3Ks are the most widely implicated in human cancers [2,14,135-138] and for this reason they will be the only PI3Ks discussed in detail in this review. Class I PI3Ks are divided further into A and B subtype. Class IA PI3Ks are dimers comprising a regulatory (p85-alpha, p85-beta, p55-alpha, p55-gamma, p50-alpha) and a catalytic (p110-alpha, p110-beta, p110-delta) subunit. Class IA PI3Ks act downstream of both tyrosine kinase receptors (TKRs) and G protein-coupled receptors (GPCRs). The single class IB PI3K comprise a p110-gamma catalytic subunit which binds one of two related regulatory subunits, p101 and p87. Class IB PI3Ks are activated downstream of GPCRs [2,14,135-138]. PI3K serves to phosphorylate a series of membrane phospholipids including: phosphatidylinositol 4-phosphate and phosphatidylinositol 4,5-bisphosphate, catalyzing the transfer of ATP-derived phosphate to the D-3 position of the inositol ring of membrane phosphoinositides, thereby forming the second messenger lipids phosphatidylinositol 3,4-bisphosphate (PIP2) and phosphatidylinositol 3,4,5-trisphosphate (PIP3) [2,14,135-138]. Most often, PI3K is activated via the binding of a ligand to its cognate receptor, whereby p85 associates with phosphorylated Y residues on the receptor via a Src-homology 2 (SH2) domain. After association with the receptor, the p110 catalytic subunit then transfers phosphate groups to the aforementioned membrane phospholipids [2,14,135-138]. It is these lipids, specifically PIP3, that attract a series of kinases to the plasma membrane thereby initiating the signaling cascade [2,14,135-138,142]. The p85 PI3K subunit also plays key roles in regulating flux through this pathway by controlling both PI3K p110 and PTEN [143].

Downstream of PI3K is the primary effector molecule of the PI3K signaling cascade, Akt/ protein kinase B (PKB) which is a 57 kDa S/T kinase that phosphorylates many targets on RxRxxS/T (R = Arginine) consensus motifs [1-3, 135-138, 142-148]. Driver *AKT* mutations are detected in some human cancer [10,49].

Akt was discovered originally as the cellular homologue of the transforming retrovirus AKT8. It is a kinase with properties similar to protein kinases A and C [2,144]. Akt contains an amino-terminal pleckstrin homology (PH) domain that serves to target the protein to the membrane for activation [1-3,135-138,142-148]. Within its central region, Akt has a large kinase domain and is flanked on the carboxy-terminus by hydrophobic and proline-rich regions. Akt-1 is activated via phosphorylation of two residues: T308 and S473, Akt-2 and Akt-3 are highly related molecules and have similar modes of activation. Akt-1 and Akt-2 are ubiquitously expressed while Akt-3 exhibits a more restricted tissue distribution. Akt-3 is found abundantly in nervous tissue [144-148].

The phosphotidylinositide-dependent kinases (PDKs) are responsible for activation of Akt. PDK1 is the kinase responsible for phosphorylation of Akt-1 at T308 [144]. Akt-1 is also phosphorylated at S473 by the mammalian target of Rapamycin (mTOR) complex referred to as (Rapamycin-insensitive companion of mTOR/mLST8 complex) mTORC2 [135-138]. Before the discovery of the ability of mTORC2 to phosphorylate S473, the activity responsible for this phosphorylation event was referred to as PDK2. Akt-2 and Akt-3 are phosphorylated in similar fashions. Therefore, phosphorylation of Akt is complicated as it is phosphorylated by a complex that lies downstream of activated Akt itself [1,2,135-138]. Thus, as with the Ras/Raf/MEK/ERK pathway, there are feedback loops that serve to regulate the activity of the Ras/PI3K/PTEN/Akt/mTOR pathway. These events also serve to illustrate that these signal transduction pathways are not really linear, but highly interactive. Once activated, Akt leaves the cell membrane to phosphorylate intracellular substrates. Akt activity is regulated by many mechanisms including the levels of PIP3 which are controlled positively and negatively by PI3K of PTEN respectively, by phosphorylation by PDK1 and mTORC2 as well as ubiquitination [149].

After activation, Akt is able to translocate to the nucleus [1-3, 134-138] where it affects the activity of a number of transcriptional regulators. Some examples of molecules which regulate gene transcription that are phosphorylated by Akt include: CREB [149], E2F [150], nuclear factor kappa from B cells (NF-kappaB) via inhibitor kappa B protein kinase (I-kappaK) [151], the forkhead (FOXO) transcription factors [152], and murine double minute 2 (MDM2) which regulates p53 activity [1-3,134-138]. These are all either direct or indirect substrates of Akt and each can regulate cellular proliferation, survival and some can modulate EMT (*e.g*., NF-kappaB) [135,151-154]. Besides transcription factors, Akt targets a number of other molecules to affect the survival state of the cell including: the pro-apoptotic molecules Bcl-2-associated death promoter (BAD) and Bcl-2 interacting mediator of cell death (Bim) [153], as well as, glycogen-synthase kinase-3beta (GSK-3beta) (see Figure 3) [164]. GSK-3beta regulates beta-catenin protein stability, which is important in regulation of EMT. When Akt phosphorylates GSK-3beta, it is targeted to the proteasome and beta-catenin is active and able to stimulate gene expression. Hence the PI3K/PTEN/Akt/mTOR pathway is connected to the Wnt/beta-catenin, p53 and many additional pathways including Ras/Raf/MEK/ERK.

Akt has many diverse effects on proliferation, survival, senescence, invasion, metastasis, drug resistance and DNA damage repair and autophagy [155-162]. Akt is involved in cell cycle progression and migration [163]. Akt may also affect the ability of miRNAs to target their respective genes. Akt is a known inhibitor of autophagy and inhibition of Akt by certain tumor suppressors will induce autophagy [164]. A recent study suggests that Akt may regulate the processing of certain miRNAs by post-transcriptional mechanisms regulate miRNAs’ processing or their stability which induces rapid fluctuation in their levels [165]. Akt and its downstream targets (*e.g*., the Foxo transcription factor) are involved in aging and suppression of Akt activity, which results in increased Foxo activity, by food supplements such as curcumin, prevents aging [166].

Negative regulation of the PI3K pathway is primarily accomplished through the action of the PTEN tumor suppressor protein. PTEN encodes a lipid and protein phosphatase whose primary lipid substrate is PIP3 [1-3,136-138,167-170]. The purported protein substrate(s) of PTEN are more varied, including focal adhesion kinase (FAK), the Shc exchange protein and the transcriptional regulators ETS-2 and Sp1 and the platelet-derived growth factor receptor (PDGFR) [167-171]. Both the lipid and protein phosphatase activities of PTEN are important for prevention of invasion [171].

PTEN has four primary structural domains. In the amino terminus is the lipid and protein phosphatase domain. This is adjacent to the C2 domain that is responsible for lipid binding and membrane localization. Next are two protein sequences rich in proline (P), glutamic acid (E), S, and T (PEST) domains that regulate protein stability. Lastly, PTEN has a PDZ domain [PDZ is an abbreviation for the first three proteins identified to share this domain which are post synaptic density protein 95 (PSD^95^), *Drosophila* disc large tumor suppressor (Dlg^1^), and zonula occludens-1 protein (zo-1)], which helps facilitate protein-protein interactions. Mutations within the phosphatase domain have been reported to nullify the endogenous function of PTEN [1,3,167]. Thus PTEN is an enticing therapeutic target for activation since it is frequently inactivated in many human cancers through point mutations as well as other genetic and biochemical mechanisms [*e.g*., promoter hypermethylation, gene deletion, expression of various interacting proteins, miRNAs, phosphorylation, acetylation, ubiquitination, oxidization and others]. PTEN inactivation results in elevated Akt activity and abnormal growth regulation [1,3,167,172]. Thus, drugs reactivating PTEN could potentially be useful in the therapy of some types of tumors driven by PTEN inactivation.

Another negative regulator of the PI3K pathway is the PH domain leucine-rich repeat protein phosphatase (PHLPP). *PHLPP* is a tumor suppressor gene. PHLPP dephosphorylates S473 on Akt-1 which can induce apoptosis and inhibits tumor growth [173]. Two other phosphatases, SHIP-1 and SHIP-2, remove the 5-phosphate from PIP3 to produce PIP2 [174-177]. *SHIP1* and *SHIP2* are tumor suppressor genes. Mutations in these phosphatases, which eliminate their activity, can lead to tumor progression.

Next we discuss some of the key targets of Akt that can also contribute to abnormal cellular growth and are key therapeutic targets [1-3,135-138,178-185]. Akt-mediated regulation of mTOR activity is a complex, multi-step phenomenon. Akt inhibits tuberous sclerosis 2 (TSC2 or tuberin) function through direct phosphorylation [179]. TSC2 is a GAP that functions in association with TSC1 to inactivate the small G protein Ras homolog enriched in brain (Rheb) [180-182]. *TSC1* and *TSC2* are both tumor suppressor and gatekeeper genes [186,187]. TSC2 has been recently shown to have other roles, for example when it interacts with transforming acidic coiled-coil-3 (TACC3) a centromere binding protein, it maintains nuclear membrane structure and regulates cell division. [188].

TSC2 phosphorylation by Akt represses GAP activity of the TSC1/TSC2 complex, allowing Rheb to accumulate in a GTP-bound state. Rheb-GTP then activates, through a mechanism not yet fully elucidated, the protein kinase activity of mTOR which complexes with Raptor (Regulatory associated protein of mTOR) adaptor protein, DEP domain containing mTOR-interacting protein (DEPTOR) and mLST8, a member of the Lethal-with-Sec-Thirteen gene family, first identified in yeast, FK506 Binding Protein 38 (FKBP38) and proline-rich Akt substrate 40 kDa protein (PRAS40) [134-138]. Raptor has also recently been shown to have other roles, including interactions with the rDNA transcriptional apparatus in the nucleoli [189].

mTORC1 inhibits Akt via a negative feedback loop which involves, at least in part, p70S6K [181]. This is due to the negative effects that p70S6K has on IRS-1 [134-138]. p70S6K phosphorylates IRS-1 on S312 and/or S636/S639. This targets IRS-1 to the proteasome where it is degraded. Hence PI3K/Akt signaling downstream of IRS-1 is downregulated when p70S6K is active. Rapamycin treatment blocks mTORC1 and p70S6K activation, thus this loop is broken and Akt is activated.

Deptor is another component of the mTORC1 complex. *DEPTOR* may be a tumor suppressor gene as decreased expression of DEPTOR results in increased mTORC1 activity [190].

The mechanism(s) by which Rheb-GTP activates mTORC1 have not been fully elucidated, however it requires Rheb farnesylation and can be blocked by farnesyl transferase (FT) inhibitors. It has been proposed that Rheb-GTP would relieve the inhibitory function of FKBP38 on mTOR, thus leading to mTORC1 activation [182].

As stated previously, TSC1 and TSC2 have important roles in the regulation of mTORC1. An additional molecule important in this regulation is the liver kinase B (LKB1 also known as STK11). *LKB1* is an important tumor suppressor and gatekeeper mutations of *LKB1* cause the rare Peutz-Jeghers Syndrome (PJS) which is a cancer-prone syndrome [191]. LKB1 is a gatekeeper gene and mutations in LKB1 are involved in the formation of hamartomatous polyps in PJS patients. LKB1 is an upstream activator of 5'AMP-activated protein kinase (AMPK) which activates TSC2 that negatively regulates mTORC1 [192,193]. LKB1 is a critical regulator of cell polarity and energy/metabolism control and exerts it vast effects via diverse effectors [138,194,195].

AMPK is considered a metabolic gatekeeper important in many diseases including diabetes, cancer and neurologic disorders [196-203]. AMPK is activated by the diabetes drug metformin [193]. Hence metformin will indirectly suppress mTORC1 activity. Chronic overfeeding increases mTORC1 activity which in turn promotes adiposity and decreases lifespan and is also believe to enhance cancer growth [204,205]. Inhibiting mTORC1 activity by drugs such as metformin and other drugs (including rapamycin) may not only aid in the treatment of diabetics, but also improve cancer therapies and increase longevity [206-216].

Akt also phosphorylates PRAS40, an inhibitor of mTORC1, and by doing so, it prevents the ability of PRAS40 to suppress mTORC1 signalling (recently reviewed in [135-138]). Thus, this could be yet another mechanism by which Akt activates mTORC1. Moreover, PRAS40 is a substrate of mTORC1 itself, and mTORC1-mediated phosphorylation of PRAS40 prevents inhibition of additional mTORC1 signaling [135-138,181]. Due to its negative regulation of mTORC1, PRAS40 has been proposed to have gatekeeper anti-apoptotic functions [217]. Also Ras/Raf/MEK/ERK signaling positively impinges on mTORC1. Both p90^Rsk-1^ and ERK 1/2 phosphorylate TSC2, thus suppressing its inhibitory function [135-138,181]. Moreover, mTORC1 inhibition resulted in ERK 1/2 activation, through p70S6K/PI3K/Ras/Raf/MEK [183].

The relationship between Akt and mTOR is further complicated by the existence of the mTOR/Rictor complex (mTORC2), which, in some cell types, displays rapamycin-insensitive activity. mTORC2 is comprised of rapamycin insensitive companion of mTOR (Rictor), mTOR, DEPTOR, mLST8, Stress activated protein kinase INteracting protein 1 (SIN1) and protein observed with Rictor (Protor). mTORC2 phosphorylates Akt on S473 *in vitro* which facilitates T308 phosphorylation [185]. Thus, mTORC2 can function as the elusive PDK-2 which phosphorylates Akt-1 on S473 in response to growth factor stimulation [186,218]. Akt and mTOR are linked to each other via positive and negative regulatory circuits, which restrain their simultaneous hyperactivation through mechanisms involving p70S6K and PI3K [135-138,181]. Assuming that equilibrium exists between these two complexes, when the mTORC1 complex is formed, it could antagonize the formation of the mTORC2 complex and reduce Akt activity [180,181]. Thus, at least in principle, inhibition of the mTORC1 complex could result in Akt hyperactivation. This is one problem associated with therapeutic approaches using rapamycin or modified rapamycins (rapalogs) that block some, but not all, actions of mTOR.

mTOR is a 289-kDa S/T kinase. mTOR was the first identified member of the phosphatidylinositol 3-kinase-related kinase (PIKK) family [135-138,219]. Recently mTOR has been shown to be cell cycle regulated [220,221]. mTOR has been referred to as the gatekeeper of autophagy. mTOR plays important roles in many biological processes, including; energy control [222-224], insulin resistance [225], diabetes [226], seizures [227,228], protein homeostasis [229], regulation of tRNA expression [230,231], cell cycle arrest [232], cell differentiation [233,234], cell migration [235,236], follicle development [237], DNA damage checkpoint [238], cellular quiescence/senescence [239-248], cancer [249,250], aging [251-260] and Parkinson's disease [261].

mTORC1 is a repressor of autophagy, a lysosome-dependent degradation pathway which allows cells to recycle damaged or superfluous cytoplasmic content, such as lipids, proteins, and organelles [262-280]. As a consequence, cells produce metabolic precursors for macromolecular biosynthesis or ATP generation. In cancer cells, autophagy fulfills a dual role, as it has both tumor-promoting and tumor-suppressing properties. Functional autophagy prevents necrosis and inflammation, which can lead to genetic instability. However, autophagy might be important for tumor progression by providing energy through its recycling mechanism during unfavorable metabolic circumstances, which are very common in tumors [262-280].

A model has been proposed by Dr. Michael P. Lisanti and colleagues which is called the reverse Warburg Effect. This model proposes that the aerobic glycolysis occurring in the tumor associated fibroblasts and not in the actual epithelial tumor cells [266,270-274]. This results in the transfer of high-energy metabolites (lactate and pyruvate) to adjacent epithelial cancer cells which fuel the cancer cells allowing them to invade and metastize. In addition, oxidative stress generated by the cancer cells induces autophagy of the tumor associated fibroblasts which the cancer cells then recycle and use to fuel their growth. Anti-oxidants (N-acetyl cysteine, NAC), quercetin and the anti-diabetes drug metformin) or autophagy inhibitors (chloroquine) will suppress the destruction of caveolin-1 in stromal fibroblasts and inhibit cancer growth. Caveolin-1 is a key protein at the cell membrane which serves to organize other important signaling molecules into signaling complexes (*e.g*., Fak, Src). Decreased expression of caveolin-1 is associated with a poorer prognosis of breast and other cancers.

Autophagy is also important in hematopoietic cancer [275-277]. Autophagy can be regulated by epigenetic mechanisms [278]. Autophagy may also become defective in certain drug resistant cells [279]. Defective autophagy may be controlled by the p53 rheostat in cancer [280]. Clearly autophagy is a very important survival process which is regulated in part by mTORC1.

mTOR regulates translation in response to nutrients and growth factors by phosphorylating components of the protein synthesis machinery, including p70S6K and eukaryotic initiation factor (eIF)-4E binding protein-1 (4EBP-1), the latter resulting in release eIF-4E, allowing eIF-4E to participate in the assembly of a translational initiation complex [1-3,135-138]. p70S6K phosphorylates the 40S ribosomal protein S6, (rpS6), leading to translation of “weak” mRNAs [1-3,135-138]. Integration of a variety of signals (mitogens, growth factors, hormones) by mTOR assures cell cycle entry only if nutrients and energy are sufficient for cell duplication [1-3,135-138].

Unphosphorylated 4E-BP1 interacts with the cap-binding protein eIF4E and prevents the formation of the 4F translational initiation complex (eIF4F); by competing for the binding of eukaryotic initiation factor 4G (eIF4G) to eIF4E. 4E-BP1 phosphorylation by mTORC1 results in the release of the eIF4E, which then associates with eIF4G to stimulate translation initiation [1-3,135-138].

eIF4E is a key component for translation of 5' capped mRNAs, that include transcripts encoding proliferation and survival promoting proteins, such as c-Myc, cyclin D1, cyclin-dependent kinase-2 (CDK-2), signal activator and transducer of transcription-3 (STAT-3), ornithine decarboxylase, survivin, B-cell lymphoma 2 (Bcl) -2, Bcl-xL, myeloid cell leukemia-1 (Mcl-1) and others [1-3,135-138].

The mechanisms which control mTORC2 activity have only begun to be revealed. mTORC2 activation requires PI3K, as inhibition of PI3K decreases mTORC2 activity [138]. mTORC2 phosphorylates Akt-1 on S473 that enhances subsequent Akt phosphorylation on T308 by PDK1. mTORC2 phosphorylates other members of the family of protein kinase A, G, and C (AGC) including as serum/glucocorticoid-regulated kinase (SGK1) [281]. mTORC2 has been shown to phosphorylate certain protein kinase C (PKC) family members [282]. mTORC2 has important roles in regulation of cell growth and it is a critical biological sensor [283]. For mTORC2 activity, it requires association with the ribosome and this may a critical sensor promoting growth when conditions are favorable but hindering growth when conditions are not favorable [284-287]. mTORC2 influences actin cytoskeletal organization [288]. Along these same lines, mTORC2 has been implicated in various aspects of tumor progression including motility, invasion and metastasis [289].

PI3K, Akt, and mTORC1/2 are linked to each other via regulatory feedback loops, which restrain their simultaneous hyperactivation. Negative regulation of Akt activity by mTORC1 is dependent on p70S6K-mediated phosphorylation of IRS-1/2 adapter proteins, downstream of the IR and/or IGF-1R [290-292]. IRS-1 and IRS-2 are normally required to activate class IA PI3Ks after stimulation of IR and IGF-1R tyrosine kinase activity. When mTORC1 is active, p70S6K phosphorylates the IRS-1/2 proteins on serine residues, targeting them for proteasomal degradation [293,294].

Inhibition of mTORC1 signaling by rapamycin/rapalogs removes the previously mentioned negative feedback loop and activates Akt through PI3K. Inhibiting mTORC1 with rapamycin will in some situations activate mTORC2. Recent findings have also highlighted the existence of a rapamycin-sensitive, mTORC1/p70S6K-mediated phosphorylation of Rictor on T1135. This phosphorylation event exerted a negative regulatory effect on the mTORC2-dependent phosphorylation of Akt *in vivo* [295]. Thus, both mTORC1 and mTORC2 could control Akt activation. PI3K/Akt/mTOR signaling is tightly controlled and negatively regulated by several lipid and protein phosphatases. PTEN removes the 3'-phosphate from PIP3, thereby antagonizing network signalling [296,297]. Two other lipid phosphatases, SHIP-1 and -2, remove the 5-phosphate from PIP3 to yield PIP2 [298].

PP2A downregulates Akt activity directly, by dephosphorylating it at T308 and accumulating evidence indicates that PP2A acts as a tumor suppressor [299]. PP2A is an essential phosphatase critically involved in regulation of cell cycle progression [300] and DNA damage response [301] as well as p53 stability and other important biochemical events.

Recent findings have indicated that there exists an inverse relationship between the levels of the B55-alpha regulatory subunit of the PP2A phosphatase, that functions as an Akt phosphatase [302] and the levels of T308 (but not S473) Akt phosphorylation levels in AML blast cells [303]. This finding suggested that B55-alpha is mediating dephosphorylation of Akt at T308, but not S473, in AML cells [303]. Interestingly, this study reported lower levels of the PP2A B55-alpha regulatory subunit in AML primary cells when compared with CD34^+^ bone marrow cells from healthy donors. Another report has documented that PP2A activity downregulation is a recurrent event in AML patients [304]. Moreover, the phosphorylated S473 residue on Akt is dephosphorylated by the two isoforms of PHLPP (1 and 2) Decreased PHLPP activity has been linked to specific types of cancers [305,306].

mTOR also controls the translation of hypoxia-inducible transcription factor-1-alpha (HIF-1-alpha) mRNA [2,26,135-138,307]. HIF-1-alpha upregulation leads to increased expression of angiogenic factors such as VEGF and PDGF which are important in many physiological processes including, blood supply, cancer and diabetes [26,308-310]. Moreover, HIF-1-alpha regulates the glycolytic pathway by controlling the expression of glucose-sensing molecules including glucose transporter (Glut) 1 and Glut3 [311,312]. p70S6K and 4E-BP1 also control cell growth and hypertrophy by regulating protein synthesis. Hence targeting the mTOR pathway could have many effects on the regulation of cellular growth.

### Mutations or Altered Expression of the Ras/PI3K/PTEN/Akt/mTOR Pathways Can Alter Sensitivity to Therapy

Mutations resulting in activation of the Ras/PI3K/PTEN/Akt/mTOR pathways and play critical roles in EMT, tumor progression and aging [313-319]. Mutations/gene amplification of *RAS*, *PIK3CA*, *PIK3R1*, *PIK3R4*, *PIK3R5*, *IRS4*, *PTEN*, *AKT1*, *TSC1*, *TSC2, RHEB, MTOR*, and *70S6K* are detected in certain cancers [5,320]. Aberrant activation of this pathway may be a contributing factor to transformation of diverse types of cancers [321]. *PIK3CA* is mutated in approximately 25% of breast, 32% of colorectal, 30% of endometrial, 27% of brain, 25% of gastric, 4% of lung cancers [322-326]. These mutations are clustered in small hot-spot regions within the helical (E542, E545) and kinase (H1047) domains [322-326]. The locations of these mutations have been recently critically evaluated [326]. These mutations frequently result in activation of its kinase activity [326]. Furthermore increased expression of the Ras/PI3K/Akt/mTOR pathway also occurs frequently in some cancers as the *PIKC3A* gene is amplified in approximately 40% of ovarian cancers [325].

Activation of PI3K/PTEN/Akt/mTOR signaling through mutation, inactivation or silencing of pathway components occurs in various malignancies, including liver cancer [327]. Deregulation of this pathway has clinical importance in hepatocellular carcinoma (HCC). For example, data from genomic sequence of HCC samples identified mutations in *PIK3CA* in 50% of patients with poor prognosis, survival length < 3 years following partial liver resection, and only 10% of the HCC patients with a good prognosis had mutations in *PIK3CA* [327]. The identified mutations were restricted to residues H1047 in 61.1%, to E545 in 33.3%, and to E542 in 5.5% of cases, and as a consequence this result in gain of enzymatic function and consequently in oncogenic activity of PI3K [327].

### Mutations at *PTEN* in Human Cancer

Germline PTEN mutations are present in approximately 80% of patients with Cowden syndrome [328]. This disease, which is also known as multiple hamartoma syndrome, is a familial syndrome that includes diverse types of cancer conditions including early onset breast cancer. Mutations have been reported to occur at *PTEN* in breast cancer in varying frequencies (5-21%) [329,330]. Loss of heterozygosity (LOH) is probably more common (30%) [330]. Mutations at certain residues of PTEN, that are associated with Cowden's disease, affect the ubiquitination of PTEN and prevent nuclear translocation. These mutations leave the phosphatase activity intact [331]. Inhibition of PTEN activity leads to centromere breakage and chromosome instability [332]. Thus PTEN has diverse activities.

Akt and mTOR phosphorylation are frequently detected in ovarian and endometrial cancers. An early occurrence in endometrial cancer is the loss of functional PTEN activity by mutation or other mechanisms, this occurs in approximately 40-80% of patients [333]. Since the loss of PTEN results in activation of Akt, that in turn up-regulates mTOR activity, cancer cells deficient in PTEN are thought to be major targets of mTOR inhibitors.

Alterations in PTEN expression have also been implicated in HCC. The best evidence that strongly supports the connection between PTEN-suppression and liver carcinogenesis comes from genetic studies. All mice with *PTEN*-deficient hepatocytes exhibited liver adenomas and 66% of them developed HCC [334]. In these mice, hepatocytes were hyperproliferative and displayed an abnormal activation of Akt [334]. Furthermore, although mutations in the *PTEN* gene rarely occur in HCC, frequent loss of heterozygosity of *PTEN* allele has been identified in 20-30% of HCC patients [335-338]. In addition, down-regulation of PTEN expression may be partly due to *PTEN* promoter methylation [339]. PTEN expression plays a critical role in HCC progression and patient's outcome. Patients with high expression of PTEN had a significantly better overall survival than patients with low PTEN expression [340]. Hepatitis viruses protect hepatocytes from apoptotic cell death by promoting the activation of Ras/PI3K/Akt/mTOR survival pathway [341,342]. Among the four proteins encoded by HBV genome, HBx has been reported to be involved in hepatocarcinogenesis. It has been reported that HBx expression downregulated PTEN expression in hepatocytes [342]. In contrast, PTEN expression in liver cells downregulated HBx-induced PI3K and Akt activities [343]. Therefore, these studies suggest the possible use of PTEN as a target in therapeutic approaches for the treatment of at least those HCC caused by HBV infection.

Mutations and hemizygous deletions of *PTEN* have been detected in AML and non Hodgkin's lymphoma (NHL) and other cancers [344,345]. Although many groups have investigated the PTEN-phosphorylation status in leukemia and lymphoma, its relevance concerning Akt-activation is still not clear [344-348]. PTEN phosphorylation as well as low or absent PTEN expression has been observed in AML.

The level of PTEN expression does not always correlate with the degree of phosphorylation of Akt [344]. Although the picture concerning PTEN-inactivation and corresponding Akt-activation is not clear, *in vivo* studies indicate, that PTEN dysregulation promotes leukemogenesis. *PTEN*-deficient hematopoietic stem cells display dysregulated cell cycle progression, and the mice develop a myeloproliferative disease which leads to leukemic transformation [346]. In T-acute lymphoblastic leukemia (T-ALL), PTEN-downregulation is also closely correlated with Akt-activation [347,348]. To discern the role of PTEN for Akt-activation, it may be useful to exclude concomitant causes for Akt-activation such as mutant upstream targets and to include the investigation of regulators of PTEN such as c-Myc and Notch/Hes1 [347,348].

PTEN promoter methylation leads to low PTEN expression [349]. In one study, 26% of primary breast cancers had low PTEN levels that correlated with lymph node metastases and poor prognoses [350].

Other mechanisms important in the regulation of PTEN are miRNAs. Certain miRNAs have been shown to regulate PTEN protein expression. mi-214 induces cell survival and may contribute to oncogenesis and drug resistance (see below) by binding the 3'untranslated region (3'UTR) of PTEN which prevents PTEN mRNA translation and leads of overexpression of downstream Akt [351].

In some cancer settings, *PTEN* and *BRAF* mutations appear to interact. Two studies papers have highlighted the hypothesis of mutant *BRAF*- and *PTEN*-loss-driven carcinogenesis in mouse models [352,353]. In a study by Dhomen *et al*., inducible expression of B-Raf^V600E^ was sufficient to induce multiple melanocytic lesions including skin hyperpigmentation, dysplastic nevi and melanoma [352]. Tumor cells from these B-Raf^V600E^ mice displayed both melanoma growth and melanocyte senescence in this system. Approximately 70% of these mice developed melanomas that exhibited histological and molecular characteristics similar to that of human melanoma and were able to colonize the lungs in nude mice [352]. In contrast, another group of researchers generated mice that conditionally-expressed melanocyte-specific *BRAF* V600E that were only able to induce benign melanocytic hyperplasias and were unable to progress any further over a 15-20 month period [353]. However, *BRAF* V600E expression in a *PTEN* gene-silenced background led to the production of melanomas with 100% establishment, short latency and metastasis to lymph nodes and lungs. This development was prevented by the treatment of mice with either the mTOR inhibitor rapamycin or the MEK1/2 inhibitor (PD0325901). Moreover, while combination treatment with rapamycin or PD0325901 led to the reduction of established tumors, upon termination of drug treatment the melanomas reappeared most likely due to the presence of drug resistant melanoma-initiating cells in these mice. Overall, these two papers further validated the mutated B-Raf/MEK/ERK and the PI3K/Akt/mTOR pathways, as promising therapeutic targets in melanoma.

### Mutations at *SHIP* Phosphatase in Human Cancer

The SHIP-1 phosphatase has been implicated as a suppressor of hematopoietic transformation as it basically can prevent Akt-activation [354]. SHIP-1-deficient mice develop a myeloproliferative disease [355] and an inactivating point mutation (*SHIP1* V684E) has been observed in approximately one of thirty AML cases [354]. Also another mutation, *SHIP1* Q1154L, has been observed in AML, but was even less frequent (1 of 192 cases) [355]. Though some studies confirmed, that SHIP-1 is a leukemia suppressor [354,355] it is unlikely that *SHIP1* mutations are a frequent cause of Akt-activation in AML. Disruption of *PTEN* or *SHIP* activity by various genetic mechanisms could have vast effects on different processes affecting the sensitivity of different cancers to various therapeutic approaches.

### Mutations of *AKT* in Human Cancer

The roles that Akt plays in cancer are complex. Akt can be activated by genetic mutations, genome amplifications and more commonly by mutations in upstream signaling components. Amplification of Akt-2 was observed in human ovarian carcinomas [356]. Increased levels of Akt are detected in carcinomas of the breast, ovary and prostate and are associated with a poorer prognosis in comparison with tumors that do not display increased levels of expression. *AKT* is a multi-gene family that consists of *AKT1*, *AKT2* and *AKT3*. *AKT1* has been reported to be mutated in some breast, colorectal, melanoma and ovarian cancers [357-359] (see below). *AKT2* is not mutated frequently in human cancer. *AKT2* is amplified in certain cancers (*e.g*., 12.1% ovarian and 2.8% breast carcinomas) [359]. Mutation of *AKT3* has been detected in some melanoma samples [360].

*AKT1* is mutated in 2 to 8% of breast, 6% of colorectal and 2% of ovarian cancers samples examined in one study [357]. This study documented an Akt mutation that results in an E for a lysine (K) substitution at amino acid 17 (E17K) in the PH domain. Cells with this *AKT1* mutation have not been observed to have mutations at *PIK3CA*; a similar scenario is also frequently observed with *RAS* and *BRAF* mutations [361]. This *AKT1* mutation alters the electrostatic interactions of Akt-1 which allows it to form new hydrogen bonds with the natural PIP3 ligand [357]. The PH domain mutation confers many different properties to the *AKT1* gene. Namely the mutant *AKT1* gene has: 1) an altered PH domain conformation, 2) is constitutively-active, 3) has an altered cellular distribution as it is constitutively-associated with the cell membrane, 4) morphologically transforms Rat-1 tissue culture cells and 5) interacts with c-Myc to induce leukemia in E-mu-Myc mice (E-mu = Enhancer of immunoglobulin mu gene, Myc = Myc oncogene originally isolated in avian myelocytomatosis virus) [357]. This PH domain mutated *AKT1* gene does not alter its sensitivity to ATP competitive inhibitors, but does alter its sensitivity to allosteric kinase inhibitors [357]. These results demonstrate that targeting the kinase domain of Akt may not be sufficient to suppress the activity of various *AKT* genes that have mutations in the PH domain. *AKT1* and *AKT3 E17K* mutations in lung cancer have been reported to be rare [362].

### Alterations of Akt Expression in Human Cancer

Akt is often upregulated in cancer cells and its overexpression is associated with a poor prognosis. Increased expression of Akt can result from activating *PIK3CA* mutations, elimination or decrease in PTEN activity or elevated PKC-epsilon expression. Elevated Akt expression has also been associated with the pathology of pancreatic, glioma and prostate cancers [363-368].

Pancreatic cancer cells have elevated IGF-1R expression and it is well known that Akt regulates IGF-1R expression [369]. This Akt effect on IGF-1R has been suggested to be responsible for the invasiveness of pancreatic cancer cells. Active Src can also activate Akt, and both Src and Akt up-regulate IGF-1R expression in this cancer. It has been demonstrated that IGF-I is expressed in the surrounding stromal cells but not in the cancer cells. This IGF-1 expression may serve as a paracrine growth factor to activate the IGF-1R pathway and the downstream Ras/PI3K/Akt/mTOR pathway in pancreatic cells.

Cyclooxygenase-2 (COX-2) is expressed at high levels in some primary endometrial tumors and is associated with an aggressive phenotype [370]. Akt is elevated and *PTEN* is often mutated in these cancers which can result in Akt activation. NF-kappaB activation has been shown to have oncogenic effects important in the control of apoptosis, cell cycle, differentiation, cell migration and inflammation [371-383]. Akt may exert its effects through the NF-kappaB pathway and COX-2 is the regulator of this pathway. Akt regulates *COX2* gene and protein expression in endometrial cancers. This study was undertaken to examine the involvement of Akt in the regulation of NF- kappaB and COX-2 [370]. The expression of both I-kappaB and phosphorylated I-kappaB were increased in the cells containing mutant *PTEN* genes. In contrast, there was no difference in NF-kappaB protein abundance between the cell lines, which differed in PTEN gene status. I-kappaB phosphorylation by the PI3K pathway was inhibited by the PI3K inhibitors Wortmannin and LY294002. There was less NF-kappaB nuclear activity, less COX-2 expression and more apoptosis after inhibition of the PI3K pathway. Dominant negative (DN) Akt blocked I-kappaB phosphorylation and decreased COX-2 expression. In contrast, introduction of constitutively-active Akt induced I-kappaB phosphorylation and up-regulated COX-2 expression.

When *PTEN* is mutated, Akt signals via the NF-kappaB/I-kappaB pathway to induce COX-2 expression in endometrial cancer cells. COX-2 can inhibit apoptosis, increase angiogenesis, and promote invasiveness. COX-2 also promotes inflammation/immunosuppression and conversion of procarcinogens into carcinogens that contribute to tumorigenesis and a malignant phenotype. This study demonstrated that Akt signals via the NF-kappaB/I-kappaB pathway to induce *COX2* gene and protein expression in endometrial cancer [370].

Elevated Akt activity can also result in increased phosphorylation of mTOR. mTOR was found to be phosphorylated in AML blasts, along with its two downstream substrates, p70S6K and 4EBP-1, in a PI3K/Akt-dependent fashion [384]. Nevertheless, others failed to detect any relationship between PI3K/Akt signalling upregulation and p70S6K phosphorylation in AML primary cells [385]. This might occur via the Ras/Raf/MEK/ERK pathway activating mTOR via ERK phosphorylation [385]. The Ras/Raf/MEK/ERK pathway is frequently activated in AML [386-388].

Akt is activated in HCC, which results in enhanced resistance to apoptosis through multiple mechanisms [389-392]. As an example, activation of the Akt pathway suppresses transforming growth factor-beta (TGF-beta) induced apoptosis and growth-inhibitory activity of CCAAT/enhancer binding protein alpha (CEBP-alpha). Activation of Akt is a risk factor for early disease recurrence and poor prognosis in patients with HCC [390]. Several mechanisms may be responsible for the activation of Akt. The high frequency of *PIK3CA* mutations and/or its upregulation in patients with shorter survival might be responsible for the Akt hyperactivation found in HCC with poor prognosis [335-341]. Selective epigenetic silencing of multiple inhibitors of the Ras pathway seems also to be responsible for the activation of Akt found in HCC [327]. Moreover, impaired expression of PTEN is involved in the regulation of Akt activity. Activation of Akt signaling and reduced expression of PTEN has been reported in 40%–60% of human HCC cases [327,335-341]. Some well known risk factors, HBV and HCV seem to utilize the Ras/PI3K/PTEN/Akt/mTOR pathway for the control of hepatocytes survival and viral replication [391,392]. Taken together, these data suggest that Ras/PI3K/Akt/mTOR pathway may represent an important therapeutic target for the treatment of HCC among patients with differing etiologies that lead to the development of this aggressive tumor.

Increased Akt activity due to upstream mutations in growth factor receptor genes or *PIK3CA* or *PTEN* may actually render cells and patients sensitive to Akt as well as downstream mTOR inhibitors. The formation of the rapamycin-sensitive mTORC1 complex in certain cancer cells that overexpress activated Akt may be altered in comparison to cells that do not overexpress Akt. In cells that express activated Akt, Akt may phosphorylate TSC2 resulting in its inactivation. In the presence of Akt activation, the mTORC1 complex is formed and downstream p70S6K and 4E-BP1 are phosphorylated, allowing the dissociation of eIF-4E, ribosome biogenesis and protein synthesis. In contrast, in the absence of Akt activation, this complex should not be formed. Rapamycin targets this complex; hence the cells that express elevated levels of activated Akt cells may be more sensitive to rapamycin than the cancer cells that do not express high levels of activated Akt. In the cells that do not express elevated levels of activated Akt, this complex should be transiently assembled after growth factor treatment. In contrast, the assembly of the rapamycin-insensitive mTORC2 complex should be lower in the cells that express elevated levels activated Akt than in those cells that do not as there is equilibrium between the mTORC1 and mTORC2 complexes. The significance of these complex biochemical signaling events is that cancer cells that overexpress activated Akt or lack PTEN/TSC1/TSC2 expression have an Achilles heel with regards to therapeutic intervention as they are highly sensitive to rapamycin treatment.

### Mutations of *TSC1/TSC2* Genes in Human Cancer

Mutations in the tumor suppressor genes *TSC1* and *TSC2* are associated with a dominant genetic disorder, tuberous sclerosis [393,394]. Patients with mutant *TSC* genes develop benign tumors (hamartomas). In contrast to Cowden's patients who have germline mutations at *PTEN* where the patients have a high propensity to develop multiple malignancies, TSC patients rarely develop multiple malignant cancers, and if they do develop malignant cancers they are usually either RCCs or angiomyolipomas [394]. This has been hypothesized to result from a lack of activation of Akt in cells that have mutant *TSC1* or *TSC2* as mTOR activity is expressed at higher levels which results in inhibition of Akt, perhaps via the effects of p70S6K on IRS1. *TSC1* has been shown to be mutated in approximately 15% of urethelial carcinomas (bladder cancers) [394]. RCCs are very sensitive to rapamycin and rapalogs.

### Altered Expression of Components Downstream of mTOR in Human Cancer

mTOR regulates translation by phosphorylating components of the protein synthesis machinery, including p70S6K and 4E-BP1 [395, 396]. p70S6K phosphorylates the 40S ribosomal protein, rpS6, leading to active translation of mRNAs [1-3]. In contrast, 4E-BP1 phosphorylation by mTORC1 on several amino acidic residues (S37; T46; S65; T70) results in the release of the eIF4E [2]. mRNAs differ in their ability to be translated; the length and sequence of the 5' UTR largely dictates the efficiency with which an mRNA transcript will be translated. Most mRNAs contain short, unstructured GC-poor 5' UTRs and are efficiently translated. In contrast, long, GC-rich sequences in the 5' UTR often hinder the ability of the eIF-4E complex to efficiently scan and initiate translation at the start codon. These are called weak mRNAs as previously discussed. Consequently, under normal circumstances these mRNAs are not efficiently translated. However, upon Akt-mediated activation of mTOR, these latter mRNAs are highly and disproportionately translated. Interestingly, many of these weak mRNAs molecules encode oncogenic proteins involved in cell proliferation or survival (*e.g.*, c-Myc, Mcl-1, cyclin-D, VEGF and survivin). These oncogenic mRNAs are therefore tightly regulated at the translation level and their accumulation in cancer cells strongly contributes to the malignant phenotype. These proteins are often subject to the phenomenon of “oncogenic shock” so when an oncogene-addicted cell is treated with a specific inhibitor the expression of these proteins rapidly decays.

Several key proteins are overexpressed as a consequence of mTOR activation including: c-Myc [397-399], cyclin D1 [399], and VEGF [400] and others. Cyclin D1 has been reported to be overexpressed in prostate cancer xenografts and metastases [401], while early stage prostatic lesions possess much lower levels of the protein [402]. A number of reports support the notion that mTOR signaling is a prominent feature of cancer progression and aging, as recurrent tumors have altered expression of a number of molecular targets of rapamycin including the above mentioned genes which encode “weak” mRNAs [403-406]. Hence mTOR inhibitors such as rapamycin may be effective in cancer therapy.

One central molecule involved in cell growth is p70S6K which is regulated by both the Ras/PI3K/PTEN/Akt/mTOR and Ras/Raf/MEK/ERK pathways [2]. The p70S6K gene is amplified in approximately 9% of primary breast cancers and elevated levels of its mRNA transcripts are found in about 41% of the tumors [407,408]. It is known that some PTEN-deficient cells and tumors that are purported to grow in response to activated Akt are hypersensitive to mTOR inhibitors. p70S6K activity is reduced by mTOR inhibitors in PTEN-deficient cells and transgenic PTEN^+/−^ mice [409,410].

## CONCLUSIONS

In this review, we have discussed the various types of mutations which occur in the Ras/Raf/MEK/ERK and Ras/PI3K/Akt/mTOR pathways and how they can lead to cancer as well as other diseases. We discussed certain classes of genes important in cancer such as oncogenes, tumor suppressor, caretaker and gatekeeper genes. It is obvious that there are many genes which can fit into more than one category. We have introduced the concepts of driver, gatekeeper, passenger, lineage-specific and synthetic lethal mutations so that the reader will have a concept of how these different classes of mutations can contribute to cancer and have been used to identify key interacting genes. We have discussed the concepts of oncogene-addiction, oncogene-bypass and kinase-switching and how they can be important in identifying the key components involved in the growth of the cancer cell and how they may change during treatment with targeted therapy. Mutations at many of the upstream receptor genes or *RAS* can result in abnormal Raf/MEK/ERK and PI3K/PTEN/Akt/mTOR pathway activation. Hence targeting these cascade components with small-molecule inhibitors may inhibit cell growth. The usefulness of these inhibitors may depend on the mechanism of transformation of the particular cancer. If the tumor exhibits a dependency on the Ras/Raf/MEK/ERK pathway, then it may be sensitive to Raf and MEK inhibitors. In contrast, tumors that do not display enhanced expression of the Ras/Raf/MEK/ERK pathway may not be sensitive to either Raf or MEK inhibitors but if the Ras/PI3K/Akt/mTOR pathway is activated, it may be sensitive to specific inhibitors that target this pathway.

Some scientists and clinicians have considered that the simultaneous targeting of Raf and MEK by individual or dual inhibitors may be more effective in cancer therapy than just targeting Raf or MEK by themselves. This is based in part on the fact that there are intricate feed-back loops from ERK which can inhibit Raf and MEK. For example when MEK1 is targeted, ERK1,2 is inhibited and the negative feed-back loop on MEK is broken and activated MEK accumulates. However, if Raf is also inhibited, it may be possible to completely shut down the pathway. This is a rationale for treatment with both MEK and Raf inhibitors or dual inhibitors. Likewise targeting both PI3K and mTOR may be more effective than targeting either PI3K or mTOR by themselves. If it is a single inhibitor which targets both molecules, such as the new PI3K and mTOR dual inhibitors this becomes a realistic therapeutic option. Finally, an emerging concept is the targeting of two different signal transduction pathways, Raf/MEK/ERK and PI3K/PTEN/Akt/mTOR for example. This has been explored in some preclinical models as well as clinical trials. The rationale for the targeting of both pathways may be dependent on the presence of mutations in either/or both pathways or in upstream Ras in the particular cancer which can activate both pathways. The concepts of targeting these pathways is considered in more detail in an accompanying review [134].
